# Research on Improving Communication Capacity in mmWave Backhaul UAV Networks

**DOI:** 10.3390/s26092700

**Published:** 2026-04-27

**Authors:** Taisei Sugimoto, Gia Khanh Tran

**Affiliations:** Department of Electronic Engineering, Institute of Science Tokyo, 2-12-1 Ookayama, Meguro-ku, Tokyo 152-8550, Japan

**Keywords:** UAV, mmWave, optimization, PSO, backhaul, resiliency, LoS, SINR, fresnel zone

## Abstract

Millimeter-wave (mmWave) unmanned aerial vehicle (UAV) networks are a promising solution for rapidly deployable backhaul systems in urban disaster scenarios, where terrestrial infrastructure may become unavailable. Although mmWave bands provide wide bandwidth for high-capacity transmission, their strong susceptibility to blockage and beam misalignment poses significant challenges in dense urban environments, particularly under UAV positional fluctuations caused by wind. This study investigates the optimization of multi-hop mmWave UAV backhaul networks with the objective of maximizing the bottleneck link capacity. A three-dimensional urban model of the Shinjuku area in Tokyo is employed, and radio propagation is evaluated using a ray-tracing-based approach considering line-of-sight (LoS) constraints and inter-link interference. Particle Swarm Optimization (PSO) is used to determine optimal UAV placements for two- to four-hop configurations. Numerical results demonstrate that multi-hop relaying combined with directional 2 × 2 patch antennas significantly improves the minimum link capacity, enabling the target backhaul capacity of approximately 9 Gbps per link under static conditions. However, capacity degradation is observed when UAV jitter is introduced due to LoS blockage and beam misalignment. To address this issue, a jitter-aware optimization method incorporating an expanded Fresnel-zone constraint is proposed. The proposed method substantially mitigates capacity degradation under realistic positional fluctuations, resulting in more robust backhaul performance. These findings demonstrate that jitter-aware placement design is essential for realizing reliable high-capacity mmWave UAV backhaul networks in dense urban disaster environments.

## 1. Introduction

### 1.1. Research Background and Purpose

In situations where communication traffic rapidly increases due to natural disasters such as earthquakes (e.g., the Noto Peninsula earthquake [1]) or large-scale events, there is a strong demand for rapidly deployable temporary communication networks that do not rely on existing terrestrial base stations. To address this issue, non-terrestrial networks represented by low Earth orbit (LEO) satellites and high-altitude platform stations (HAPS) have attracted considerable attention, and active research has been conducted in recent years. Among these non-terrestrial networks, unmanned aerial vehicle (UAV) base stations are regarded as a promising technology due to their high mobility and flexible deployment capability. In communication networks employing UAV base stations, backhaul links formed between UAVs or between UAVs and ground stations play a crucial role, in addition to access links that provide connectivity to ground user terminals. Since backhaul links are required to aggregate and relay a large volume of communication traffic, high-speed and high-capacity transmission performance is essential. To meet this requirement, millimeter-wave (mmWave) communication, which can exploit wide frequency bandwidth, is considered a promising wireless technology [2]. However, mmWave signals are highly susceptible to blockage by obstacles such as buildings, making the acquisition of line-of-sight (LoS) challenging in dense urban environments. Furthermore, UAVs are subject to position fluctuations caused by external disturbances such as wind, which can lead to capacity degradation or link disconnection. Considering these challenges, this study focuses on an mmWave UAV backhaul network and investigates UAV placement optimization to maximize communication capacity in an urban environment with realistic building models. In particular, multi-hop relay configurations employing multiple UAVs are considered, and a communication capacity model incorporating LoS constraints and inter-link interference is developed. As a network-wide performance metric, the bottleneck capacity, defined as the minimum link capacity, is adopted, and the problem is formulated as an optimization problem that maximizes this bottleneck capacity. In addition, since rapid network deployment using a limited number of UAVs is required in disaster scenarios, this study also focuses on the minimum number of relay hops necessary to satisfy a given communication capacity requirement. Furthermore, UAV position fluctuations are modeled as spatial perturbations, and UAV placements that can maintain communication performance even under such fluctuations are evaluated. Here, the communication demand assumed in this study is quantitatively examined based on an urban disaster evacuation scenario. Specifically, a disaster occurring around Shinjuku Station is considered, where people located in both the eastern and western areas evacuate to shelters in the western Shinjuku area, which is characterized by a high density of buildings. The target urban model covers an area of approximately $2.295\;{\mathrm{km}}^{2}$, and by considering both the eastern and western areas, the total target area becomes $4.59\;{\mathrm{km}}^{2}$. The daytime population density of Shinjuku Ward is reported to be $44,184.9\;\mathrm{persons}/{\mathrm{km}}^{2}$ [3]. Therefore, the total daytime population within the target area is estimated as

$$44,184.9\times 4.59\approx 2.02\times 10^{5}\;\mathrm{persons}.$$

As a representative communication demand during disasters, voice calls using smartphones are assumed. For a typical communication application, LINE, the data volume required for one hour of voice calls is approximately 20MB [4]. Assuming a worst-case scenario in which all users simultaneously engage in voice calls, the total required data volume is estimated as

$$20\;\mathrm{MB}\times 2.02\times 10^{5}\approx 4.0\;\mathrm{TB}.$$

Assuming that this data volume is transmitted within approximately one hour, the required average communication rate *R* is given by

$$R=\frac{4.0\;\mathrm{TB}\times 8}{3600\;s}\approx 9.0\;\mathrm{Gbps}.$$

When bidirectional communication, i.e., uplink (UL) and downlink (DL), is taken into account, the total required backhaul capacity becomes approximately 18Gbps. In this study, a network configuration in which this traffic demand is shared by two backhaul links is assumed, and thus the required capacity per backhaul link is set to approximately 9Gbps.

**Figure 1:**
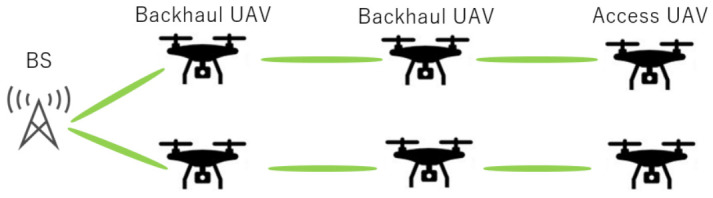
Backhaul Network System Model.

Based on the above discussion, this study aims to clarify the minimum number of relay hops required to achieve approximately 9 Gbps per link in dense urban environments, along with the corresponding UAV deployment conditions. Figure 1 shows the network configuration for this study. Here, the access UAVs are defined as UAVs that provide communication services to ground terminals such as UE, while the backhaul UAVs act as relay nodes connecting the BS and the access UAVs. The green ovals represent the communication links between nodes.

Recently, extensive research has been conducted on communication networks employing unmanned aerial vehicles (UAVs). However, several challenges remain in constructing reliable millimeter-wave (mmWave) UAV backhaul networks in dense urban environments.

First, mmWave communication is highly susceptible to blockage caused by obstacles such as buildings, making it difficult to establish line-of-sight (LoS) links in urban areas. Second, UAV platforms may experience positional fluctuations due to external disturbances such as wind, which can result in beam misalignment and communication capacity degradation. Third, in order to enable rapid network deployment in disaster scenarios, it is necessary to optimize UAV placement so that high-capacity backhaul communication can be achieved using a limited number of UAVs.

To address these challenges, this study proposes an integrated optimization framework for multi-hop mmWave UAV backhaul networks in urban environments. The proposed approach incorporates a realistic radio propagation environment based on a three-dimensional urban building model, LoS constraints, and UAV positional jitter into the UAV deployment design process.

### 1.2. Previous Research

Recently, UAV-assisted wireless communication networks have attracted significant research attention. For example, a recent study proposed an adaptive stabilization control mechanism based on deep reinforcement learning (DRL) for buoyancy-assisted autonomous aerial vehicles (AAVs) designed for long-duration hovering missions [5]. In that work, buoyancy was utilized to reduce the weight of the UAV and increase battery capacity, thereby extending the flight time. To address instability caused by strong winds and the inverted pendulum effect of the balloon structure, a DRL-based control mechanism was used to dynamically adjust rotor speeds to stabilize the UAV posture. Experimental results demonstrated that the proposed control scheme can effectively stabilize the UAV and extend the flight time compared with conventional UAV systems.

In addition, the integration of UAVs with reconfigurable intelligent surfaces (RIS) has been investigated for next-generation wireless networks [6]. In this study, the energy-efficient transmission strategy in a network consisting of multiple UAVs and beyond-diagonal RIS (BD-RIS) was optimized. Specifically, the authors formulated an optimization problem to jointly determine RIS phase rotations, UAV trajectories, rate allocation in rate-splitting multiple access (RSMA), and precoder design. The problem was modeled as a mixed-integer nonlinear programming (MINLP) problem and solved using a combination of generalized Benders decomposition, manifold-based optimization, and successive convex approximation. Simulation results showed that the proposed approach significantly improves energy efficiency in UAV-assisted communication networks.

UAVs in UAV networks have been studied from various perspectives. Reference [7] investigated the three-dimensional placement of UAV base stations to maximize the number of users covered with minimal power consumption. Reference [8] determined UAV placement by solving a particle swarm optimization problem to optimize the coverage area of UAV base stations. Reference [9] improved coverage while avoiding interference between UAV base stations using an algorithm based on simulated annealing.

Research on UAV base stations has primarily focused on examining microwave-band UAV base stations, emphasizing connectivity. However, rescue operations using remotely controlled equipment are expected to become more active in disaster areas, necessitating high-capacity transmission. Furthermore, as communication speeds increase, the size of web pages continues to grow annually [10], necessitating high-capacity transmission for information gathering in disaster areas. Therefore, in the future, when millimeter waves are routinely used, it is anticipated that microwave-band UAV base stations will struggle to address such cases. Consequently, this study employs millimeter-wave-band UAV base stations for its analysis.

In millimeter-wave communications, the existence of line-of-sight (LoS) links has a significant impact on communication capacity. Millimeter-wave signals are highly susceptible to blockage by obstacles, and under non-line-of-sight (NLoS) conditions, the propagation loss increases significantly, resulting in a substantial reduction in communication capacity. For example, Rappaport et al. demonstrated that when the LoS path is blocked in millimeter-wave communications, the received power decreases drastically and the communication performance degrades significantly [11]. Therefore, many studies on millimeter-wave UAV backhaul networks assume the establishment of LoS links as a fundamental design condition. Based on this understanding, this study performs UAV placement optimization considering Fresnel-zone constraints and UAV jitter in order to maintain stable LoS communication links and improve the reliability of high-capacity backhaul transmission.

Furthermore, many papers on UAV base station placement calculate propagation loss based on line-of-sight rates, simplifying the consideration of building blocking. Therefore, this study analyzes radio wave propagation using actual building data to optimize UAV base station placement.

Extensive research has investigated the utilization of millimeter-wave (mmWave) bands for enhancing 5G cellular networks in response to the explosive growth of mobile traffic. In particular, Sakaguchi et al. proposed a comprehensive network architecture aiming to achieve a 1000-fold increase in system throughput within ten years [12]. Their approach integrated mmWave small-cell base stations and conventional macro base stations into a multi-band heterogeneous network connected to a Centralized-RAN (C-RAN) architecture.

By adopting user-plane/control-plane splitting, the proposed architecture enables dynamic cell structuring to match the limited coverage of mmWave access with high-traffic user locations. To validate the effectiveness of this design, system-level simulations were conducted incorporating a projected future traffic model, a measurement-based mmWave propagation model, and a centralized cell association algorithm exploiting the C-RAN framework.

The numerical results demonstrated that the proposed mmWave-enabled architecture can achieve a 1000-fold increase in system rate compared to current networks, which cannot be realized by small-cell deployments operating in the commonly considered 3.5 GHz band. Furthermore, the study discussed the latest developments in mmWave devices and regulatory frameworks, highlighting the feasibility of mmWave utilization in 5G systems.

Tran et al. proposed a comprehensive framework for smart wireless aerial networks to support high-fidelity digital twin (DT) systems [13]. Their study analyzed the required throughput for airborne LiDAR point cloud transmission and developed a millimeter-wave (mmWave) aerial link budget model to meet stringent data rate requirements. A software-defined network architecture integrating Network Function Virtualization (NFV) and Software-Defined Networking (SDN) was introduced to enable dynamic UAV orchestration, routing control, and network slicing. To improve robustness and transmission efficiency, they further proposed a multi-route redundant communication framework and a semantic image transmission protocol based on deep joint source-channel coding (DJSCC) with feature-based elastic compression. Moreover, a multi-agent reinforcement learning strategy was employed to enable autonomous UAV placement and relay network formation in dynamic environments. Simulation results demonstrated the scalability and adaptability of the proposed system, highlighting its potential for reliable DT construction across diverse deployment scenarios.

Tran et al. provided a digest of the B5G white paper on artificial relaying technologies, including repeaters, metasurfaces, and RIS/IRS [14]. Their work systematically summarized the use cases, technical trends, and international standardization activities related to decode-and-forward (DF), amplify-and-forward (AF), and network-controlled reflection technologies.

In addition, they conducted experimental evaluations of mmWave analog repeaters and demonstrated the effectiveness of wireless multi-hop configurations for coverage extension. The measurement results showed that introducing multiple repeaters enabled stable downlink throughput on the order of 1 Gbps even in blocked environments. These findings highlight the importance of relay-assisted transmission techniques in overcoming propagation loss and blockage issues in high-frequency bands.

Furthermore, considering the practical operation of UAV networks, the difficulty of continuous operation stemming from UAV battery constraints is also a critical challenge. Reference [15] explicitly modeled an operation where drones depart for maintenance points at fixed intervals and return after battery replacement, targeting drone radio relay networks during large-scale disasters. This paper focused on how network topology changes during departure, affecting delay and reachability. It introduces a flight model accounting for departure movements (e.g., operations to restore network shape with remaining drones after departure) to reduce information transmission delays. It also demonstrated that dispersing departure timing to minimize fluctuations in operational drone numbers is effective, as the simultaneous departure of all UAVs temporarily reduces operational capacity significantly, increasing delays. Thus, the battery issue is addressed by modeling departure and return operations based on battery replacement and mitigating performance degradation by optimizing departure timing and maintenance site allocation.

Regarding Backhaul Networks, numerous studies on wireless communication networks using UAVs have been reported. There has been particular attention on the ability to build flexible communication networks independent of ground infrastructure by utilizing UAVs deployed in the air as base stations or relay stations. Al-Hourani et al. analyzed the relationship between the altitude of UAV base stations and coverage to ground users, proposing a probabilistic line-of-sight (LoS) model. Additionally, Mozaffari et al. conducted a systematic investigation into the deployment optimization problem for cellular networks using UAVs.

In addition, several studies have investigated realistic propagation phenomena and deployment strategies for millimeter-wave UAV communications in urban environments. For example, a recent work on an air-to-air (A2A) channel model incorporating rooftop specular reflections and UAV body blockage demonstrated that urban structures and UAV geometry significantly influence radio propagation characteristics in high-frequency bands [16]. These findings indicate that practical UAV communication systems must consider not only line-of-sight (LoS) conditions but also additional propagation effects caused by reflections and physical obstruction by the UAV platform itself.

Furthermore, research on dynamic deployment strategies for UAV-assisted millimeter-wave networks in post-disaster urban environments has emphasized the importance of adaptive network planning under emergency conditions [17]. In such scenarios, UAV relay nodes must be rapidly deployed while considering environmental constraints and communication reliability, highlighting the necessity of robust network design methods capable of maintaining stable high-capacity links.

As research on millimeter-wave backhaul, Nakamura constructed a proof-of-concept system for an SDN-controlled millimeter-wave mesh backhaul network and investigated improvements in user-perceived performance under conditions where backhaul becomes a bottleneck [18]. This research aimed to reduce switching delays and backhaul congestion-induced latency by implementing prefetching. This involved moving necessary data and processing tasks to nearby nodes in advance, before the user moves and the communication path switches. This concept of pre-placement is also useful when uplink and downlink traffic share the same link via time division multiplexing. By moving data during the available communication window beforehand, it mitigates the degradation in perceived performance caused by switching.

Researching UAV backhaul networks using the millimeter-wave band, Hirata designed a millimeter-wave mesh backhaul network employing UAVs [19]. This research focused on UAV backhaul networks using highly directional antennas in the millimeter wave band and conducted a detailed examination of network construction methods that consider base station sector division and antenna directivity. Specifically, it demonstrated through numerical analysis that appropriately positioning backhaul UAVs can reduce interference and improve the overall communication capacity of the network.

However, Hirata’s research primarily assumed ideal propagation environments and static UAV placement. It did not sufficiently account for the impact of building obstructions in urban environments or communication quality fluctuations caused by UAV vibrations. Furthermore, the evaluation metrics focused primarily on overall communication capacity. Analysis focusing on the minimum communication link capacity (bottleneck capacity), which is dominant in multi-hop configurations, is limited.

Building upon these prior studies, this research targets millimeter-wave UAV backhaul networks. It performs communication evaluations considering building models simulating urban environments and UAV vibrations. Furthermore, to appropriately evaluate the overall network performance in multi-hop configurations, this study focuses on the bottleneck capacity—defined as the minimum link capacity—and characterizes it as an UAV placement optimization problem aimed at maximizing this capacity.

The main contributions of this study are summarized as follows:

- A communication evaluation framework for multi-hop millimeter-wave UAV backhaul networks that explicitly considers urban building blockage and UAV positional jitter.
- A quantitative analysis of the impact of the number of relay hops on the bottleneck capacity in multi-hop UAV backhaul networks.
- An evaluation of millimeter-wave communication performance under UAV positional jitter, clarifying its impact on communication capacity and link stability.

## 2. Methods

### 2.1. Overall Architecture of the UAV Network

**Figure 2:**
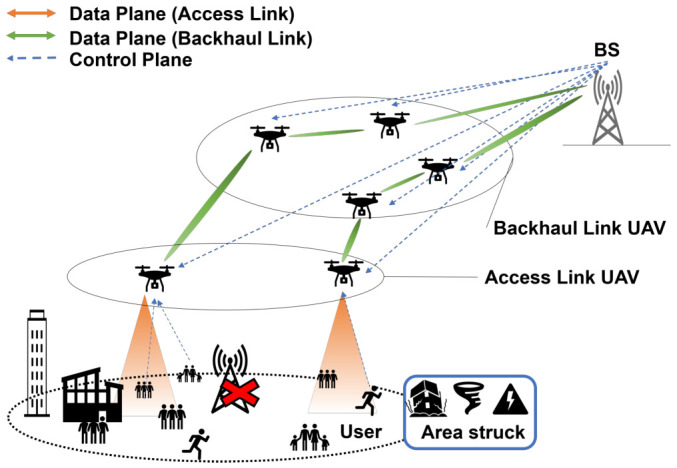
System model of the considered UAV network.

Figure 2 illustrates the overall architecture of the UAV network considered in this study. We assume a disaster scenario in which terrestrial base stations (BSs) become unavailable due to natural disasters such as earthquakes or typhoons, resulting in a sudden increase in communication traffic. Under such conditions, a rapidly deployable temporary communication network that does not rely solely on existing terrestrial infrastructure is required. Unmanned aerial vehicles (UAVs) can be flexibly deployed in three-dimensional space and can more easily maintain line-of-sight (LoS) conditions, which are essential for millimeter-wave (mmWave) communications. In addition, UAV-based networks can be deployed without installing new ground infrastructure, making them suitable for emergency and temporary communication services. The considered UAV network consists of two types of UAVs: access UAVs and backhaul UAVs. Access UAVs directly provide communication services to ground users, while backhaul UAVs act as relays that connect access UAVs to an undamaged terrestrial BS. Although mmWave communications enable high-capacity transmission, they suffer from severe distance-dependent path loss, which makes direct communication between the BS and access UAVs inefficient over long distances. To address this issue, this study adopts a multi-hop backhaul architecture using multiple backhaul UAVs to divide the communication distance and improve the backhaul capacity. In dense urban environments, building blockages often prevent LoS links and significantly degrade communication performance. Therefore, this study focuses on optimizing the placement and hop configuration of backhaul UAVs in a three-dimensional urban environment to maximize the minimum backhaul link capacity, referred to as the bottleneck capacity.

### 2.2. Simulation Environment and Evaluation Scope

**Figure 3:**
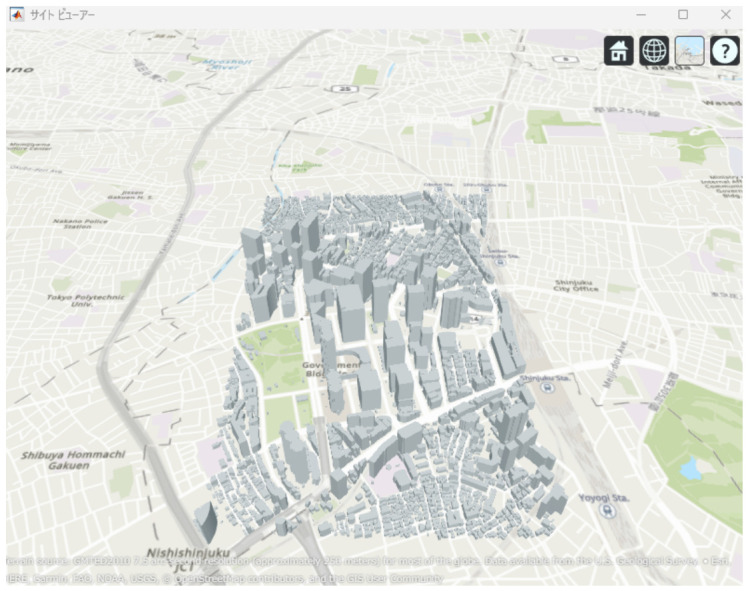
3D urban model of the Shinjuku area used for simulations.

This study evaluates a mmWave UAV backhaul network by considering the impact of building blockages in an urban environment. A three-dimensional city model of the Shinjuku area, as shown in Figure 3, is used to realistically reproduce LoS and non-line-of-sight (NLoS) conditions between UAVs and between UAVs and the terrestrial BS. In this study, access links between UAVs and ground user equipment are not considered. Only backhaul links consisting of UAV-to-UAV and UAV-to-BS communications are evaluated.

The primary objective of this study is to analyze the communication performance of a mmWave UAV backhaul network under realistic urban propagation conditions, particularly focusing on the impact of building blockage and UAV positional jitter on communication capacity. For this purpose, the evaluation in this paper considers a moderate-scale UAV network configuration based on the Shinjuku urban model. One possible large-scale deployment scenario can be envisioned in rural areas, where a relay UAV network is constructed to provide connectivity from distant terrestrial base stations. In such environments, the number of buildings is significantly smaller than in dense urban areas, which reduces the number of blockage checks in ray-tracing calculations. As a result, the computational load required for communication evaluation can be relatively smaller compared with dense urban scenarios. In contrast, in dense urban areas such as central Tokyo, the density of terrestrial base stations is relatively high. Even in disaster situations, it is unlikely that all base stations become unavailable simultaneously, and multiple surviving base stations may remain operational. In such cases, UAV backhaul networks can be constructed in a distributed manner across multiple base stations, reducing the need for extremely long multi-hop relay chains originating from a single base station. Based on these considerations, the proposed optimization framework is expected to be applicable to both urban and rural deployment scenarios.

### 2.3. Ray-Tracing Method

To accurately evaluate radio wave propagation in an urban environment, a ray-tracing method is employed. Ray tracing models electromagnetic wave propagation as rays and calculates propagation characteristics such as path loss by tracking propagation paths from the transmitter to the receiver [20]. The simulations are conducted using the RayTracing object implemented in MATLAB 2024a. This enables propagation analysis that accounts for three-dimensional building structures and their blocking effects. As the urban environment model, three-dimensional building data provided by the PLATEAU project, promoted by the Ministry of Land, Infrastructure, Transport and Tourism of Japan, are used [21]. The simulation area is set around the Shinjuku metropolitan region, which is characterized by densely packed high-rise buildings. In such environments, LoS links between UAVs or between UAVs and the BS are frequently blocked, making UAV placement a critical factor for mmWave backhaul performance.

### 2.4. Construction of the Simulation Environment

The procedure for constructing the simulation environment is summarized as follows:

(1) Import the three-dimensional building model of the Shinjuku area obtained from PLATEAU into MATLAB.
(2) Deploy one terrestrial BS and two access UAVs.
(3) Deploy multiple backhaul UAVs to form a multi-hop backhaul network.
(4) Vary the placement of backhaul UAVs to maximize the capacity of the bottleneck backhaul link.

### 2.5. System Model and Capacity Evaluation

This study considers a multi-hop UAV backhaul network in which access UAVs are connected to a terrestrial BS via multiple relay UAVs. In this study, a wide-area communication scenario in an urban environment is considered, where the distance between the terrestrial base station (BS) and the access UAV exceeds approximately 1 km. To enable communication over such relatively long distances, a multi-hop backhaul architecture using multiple relay UAVs is employed. The placement of relay UAVs is optimized using an optimization algorithm while considering line-of-sight (LoS) conditions in the urban environment. Therefore, the distances between UAVs are not predetermined but are automatically determined through the optimization process. Since the end-to-end backhaul performance is limited by the minimum capacity among all backhaul links, the bottleneck link capacity is used as the primary evaluation metric. Decode-and-Forward (DF) relaying is adopted at each backhaul UAV. In the DF scheme, the received signal is decoded and re-encoded at each hop, preventing noise accumulation across hops. The objective of this study is to achieve a target per-link backhaul capacity of 9 Gbps with the minimum possible number of relay UAVs.

### 2.6. Propagation Loss and Capacity Model

In this study, radio propagation characteristics of backhaul links between UAVs and between UAVs and a terrestrial base station (BS) are modeled, and the corresponding communication capacity is evaluated. The path loss of each link is represented as the sum of free-space path loss and additional losses caused by the surrounding environment, and it takes different values depending on whether the link is in a line-of-sight (LoS) or non-line-of-sight (NLoS) condition. The path loss $L_{\mathrm{LoS}/\mathrm{NLoS}}$ is defined as follows:

(1) $$L_{\mathrm{LoS}/\mathrm{NLoS}}\left[\mathrm{dB}\right]=L_{\mathrm{free}}+\eta_{\mathrm{LoS}/\mathrm{NLoS}}$$

Here, $L_{\mathrm{free}}$ denotes the free-space path loss, and $\eta_{\mathrm{LoS}/\mathrm{NLoS}}$ represents the additional loss that accounts for environmental effects such as building blockages in urban areas. The free-space path loss $L_{\mathrm{free}}$ is given by

(2) $$L_{\mathrm{free}}\left[\mathrm{dB}\right]=20\log_{10}d+20\log_{10}f+20\log_{10}\frac{4\pi }{c}$$

where *d* is the distance between the transmitter and the receiver, *f* is the carrier frequency, and *c* is the speed of light.

Based on Friis’ transmission equation, the received signal power $P_{r}$ is calculated as

(3) $$P_{r}\left[\mathrm{dBm}\right]=P_{t}+G_{t}+G_{r}-L$$

where $P_{t}$ denotes the transmit power, $G_{t}$ and $G_{r}$ are the gains of the transmit and receive antennas, respectively, and *L* represents the path loss of the corresponding link.

The noise power $P_{n}$ is expressed using the noise power spectral density $\sigma_{n}^{2}$ and the bandwidth *B* as

(4) $$P_{n}=\sigma_{n}^{2}B$$

Furthermore, to account for inter-link interference in a multi-hop UAV backhaul network, this study evaluates communication quality using the signal-to-interference-plus-noise ratio (SINR). The SINR is defined as

(5) $$\mathrm{SINR}=\frac{P_{r}}{P_{n}+\sum_{i}P_{\mathrm{int},i}}$$

where $P_{\mathrm{int},i}$ denotes the interference power from other links *i* operating in the same frequency band.

Based on the obtained SINR, the communication capacity $C_{i}$ of each backhaul link *i* is calculated using Shannon’s capacity [22] formula as

(6) $$C_{i}=B\log_{2}\left(1+{\mathrm{SINR}}_{i}\right)$$

where ${\mathrm{SINR}}_{i}$ represents the signal-to-interference-plus-noise ratio of link *i*.

To evaluate the overall performance of the multi-hop UAV backhaul network, the communication capacities of all backhaul links are computed, and the minimum among them is defined as the bottleneck capacity of the network. In this study, maximizing this bottleneck capacity is set as the optimization objective.

In the present study, atmospheric effects, such as rain attenuation, fog attenuation, and variations in temperature and air density, are not explicitly included in the propagation model. The main focus of this study is to evaluate the impact of UAV positional jitter on the stability of millimeter-wave UAV backhaul links in urban environments. Therefore, the analysis primarily considers geometric link stability and inter-link interference rather than detailed atmospheric attenuation modeling.

It should also be noted that the influence of environmental conditions on UAV millimeter-wave communication has been investigated in previous work. For example, Sugimoto and Tran [23] studied UAV millimeter-wave communication using hybrid sensor fusion beamforming and discussed the impact of environmental conditions on communication performance. According to the results reported in that study, for the relatively short communication distances considered in UAV backhaul links, the attenuation caused by rainfall is expected to be limited under weather conditions in which UAV operation is still feasible. Therefore, under the scenario assumed in this study, the impact of atmospheric attenuation, such as rain and fog, is considered to be relatively small.

### 2.7. Overview of the Optimization Algorithm

The communication capacity maximization problem in millimeter-wave UAV backhaul networks addressed in this study depends strongly on the three-dimensional placement of UAVs and the orientation of directional antennas. As a result, the search space becomes high-dimensional and highly complex. In particular, in urban environments, the communication conditions between UAVs or between UAVs and ground base stations vary significantly due to building blockage, and the relationship between UAV placement and communication capacity exhibits strong nonlinearity and multimodality.

Conventional studies on UAV backhaul networks often evaluate the overall network throughput under ideal propagation environments. However, practical factors such as building blockage in urban environments and communication quality fluctuations caused by UAV positional jitter are not sufficiently considered. Therefore, in this study, the UAV placement optimization problem in a multi-hop millimeter-wave UAV backhaul network is formulated as a numerical optimization problem that aims to maximize the bottleneck capacity of the network. Specifically, building models representing urban environments and UAV positional jitter are explicitly incorporated into the communication evaluation and optimization process. The objective function is defined as the minimum communication capacity among all backhaul links in the network, and the optimization procedure searches for UAV placements that maximize this bottleneck capacity.

Based on the above formulation, the objective function in this study is defined using the Shannon capacity formula. Specifically, the optimization aims to maximize the minimum communication capacity among all relay links in the network, which determines the bottleneck performance of the multi-hop UAV backhaul network. The objective function can therefore be expressed as follows:

(7) $$\max\min_{i}C_{i}=B\log_{2}\left(1+{\mathrm{SINR}}_{i}\right)$$

where $\min_{i}C_{i}$ represents the minimum link capacity among all relay links (bottleneck capacity), *B* denotes the communication bandwidth, and ${\mathrm{SINR}}_{i}$ is the signal-to-interference-plus-noise ratio of the bottleneck link, where the interference term considers the aggregate interference from all transmitting nodes in the network.

For such a problem, design approaches based on geometric rules, such as placing relay UAVs at equal intervals, cannot sufficiently account for building layouts and blockage conditions, making it difficult to uniquely determine a placement that maximizes the minimum link capacity. Furthermore, previous studies have pointed out that, in relay placement for multi-hop wireless networks, simple equal-interval placement is not optimal due to the impact of inter-link interference [24].

Furthermore, local search-based optimization methods, such as gradient-based approaches, often require the objective function to be differentiable. However, in this study, the minimum link capacity changes discontinuously depending on the presence or absence of building blockage, making it difficult to stably define gradient information. In addition, gradient-based methods are highly sensitive to initial values and tend to converge to local optima, which makes them unsuitable for UAV placement optimization that requires global exploration to improve network-wide bottlenecks.

Moreover, for nonlinear and non-convex optimization problems such as UAV placement optimization, heuristic optimization methods, including the Genetic Algorithm (GA) and Simulated Annealing (SA), can also be applied.

In the optimization problem considered in this study, the objective function is defined as the maximization of the minimum backhaul link capacity, which determines the bottleneck performance of the network. This problem can be formulated as a numerical optimization problem with a continuous-valued objective function. For such problems with continuous evaluation metrics, Particle Swarm Optimization (PSO) [25], which performs solution search based on particle position updates in the continuous search space, is considered suitable.

PSO is considered to have the tendency to converge relatively quickly toward promising solution regions in many continuous optimization problems. Although the exploration range tends to gradually shrink as the search progresses, this behavior promotes efficient convergence in optimization problems where the objective function varies continuously, as in the problem considered in this study.

**Figure 4:**
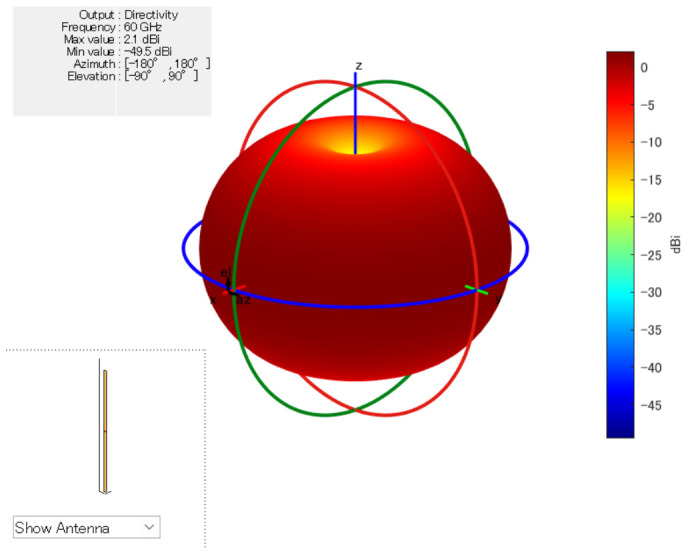
3D Antenna Pattern (Dipole Antenna).

**Figure 5:**
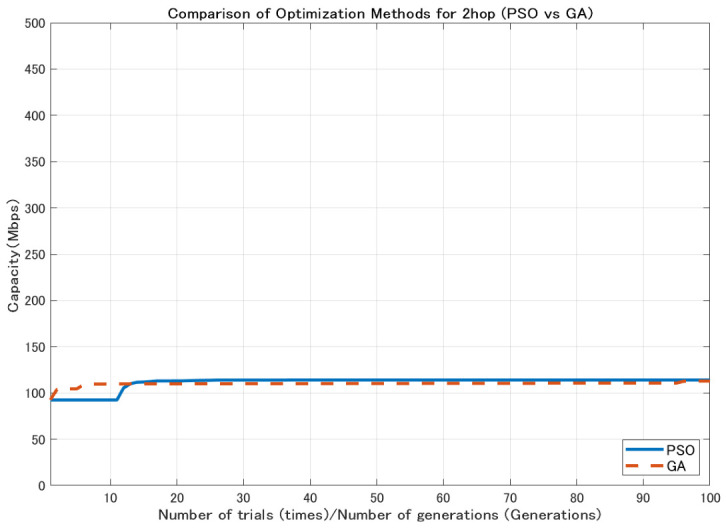
Comparison of optimization methods for 2-hop configuration.

**Figure 6:**
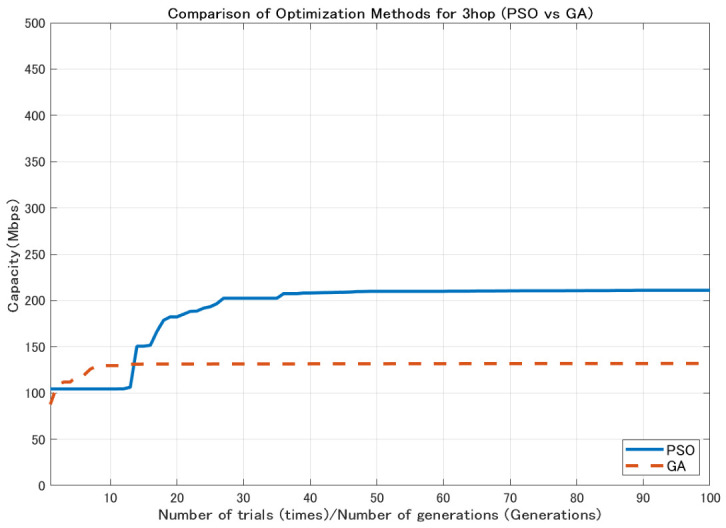
Comparison of optimization methods for 3-hop configuration.

**Figure 7:**
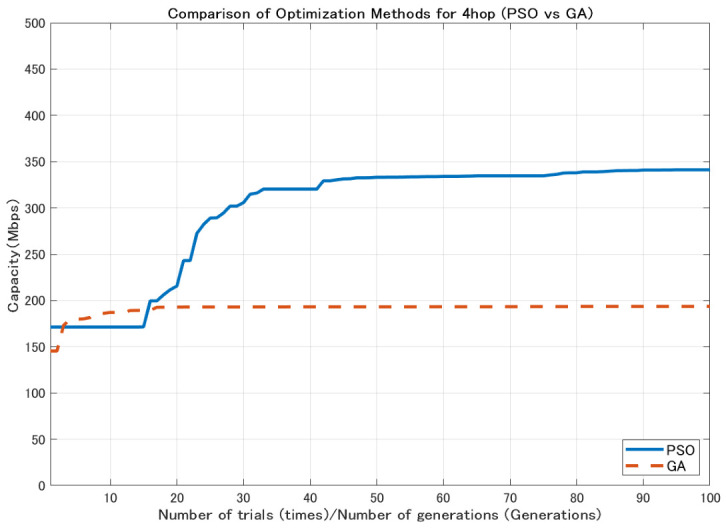
Comparison of optimization methods for 4-hop configuration.

In contrast, GA generates new candidate solutions through mutation and crossover operations, which enables wide exploration of the search space [26]. However, depending on the characteristics of the problem, a relatively large number of generations may be required to obtain high-quality solutions. Using the 2.1 dBi dipole antenna shown in Figure 4, the comparison results (Figure 5, Figure 6 and Figure 7) show that GA achieves lower minimum link capacity than PSO for all hop configurations, and exhibits less stable convergence behavior.

Similarly, SA performs stochastic updates of candidate solutions during the search process [27]. In SA, solution updates are generally performed sequentially, and therefore, a large number of iterations may be required to sufficiently explore large continuous search spaces. In addition, the performance of SA is known to strongly depend on the temperature cooling schedule, and inappropriate parameter settings may degrade the search efficiency. Therefore, for the multi-variable continuous optimization problem considered in this study, PSO is considered to be an effective approach.

For these reasons, this study employs Particle Swarm Optimization (PSO), which does not require gradient information and has strong global search capability, to solve the minimum link capacity maximization problem formulated as a continuous numerical optimization problem.

PSO is an optimization technique inspired by the collective behavior of swarming organisms. Each particle in the search space represents a set of design variables, including the three-dimensional UAV positions and antenna parameters. Particles move by referring to their own best historical solutions and the globally shared best solution, achieving convergence toward an optimal solution while balancing exploration and exploitation in the continuous search space.

**Figure 8:**
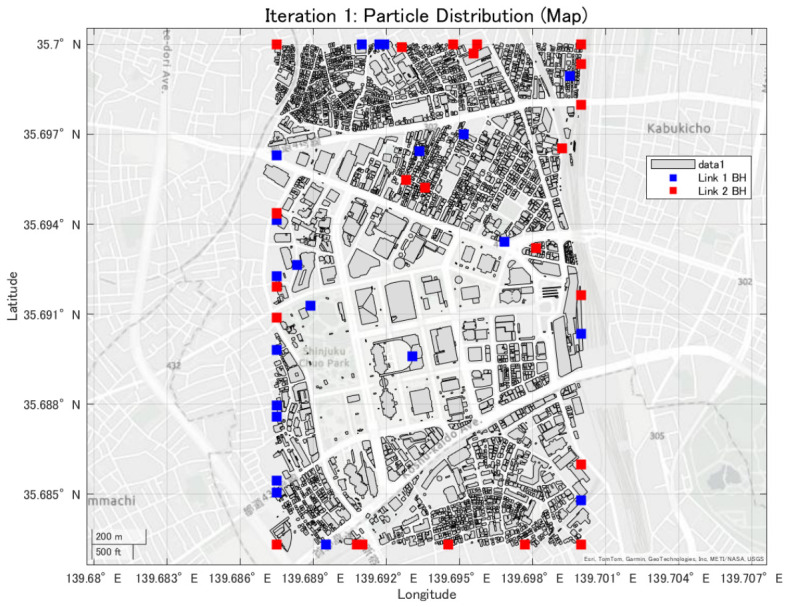
Convergence process of the best evaluation value in PSO (1th trial).

**Figure 9:**
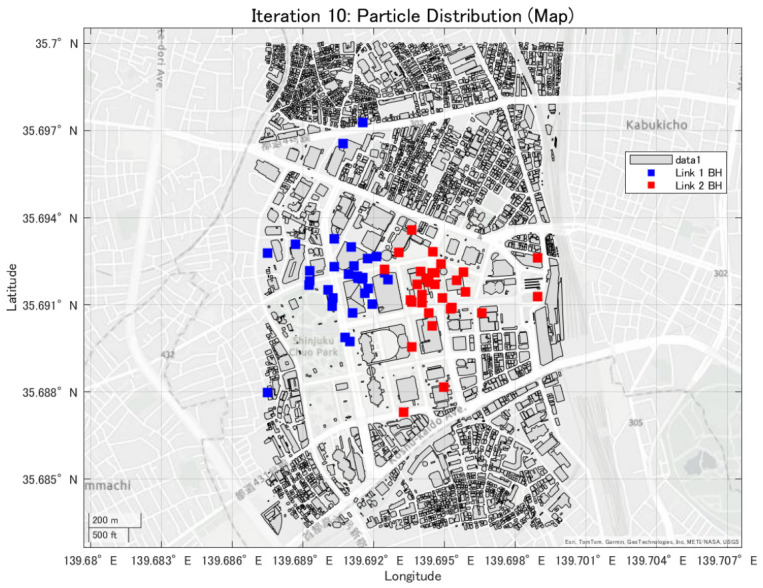
Convergence process of the best evaluation value in PSO (10th trial).

**Figure 10:**
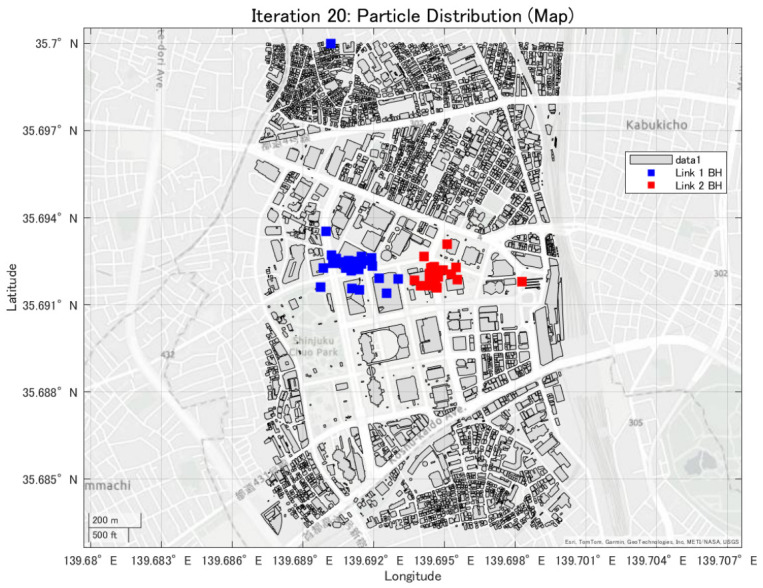
Convergence process of the best evaluation value in PSO (20th trial).

In this study, the minimum backhaul link capacity is used as the evaluation function, and the particle swarm evolves so as to maximize this continuous-valued metric. An example of the convergence behavior of PSO, showing the transition of the best evaluation value with respect to the iteration count, is presented in Figure 8, Figure 9 and Figure 10.

## 3. Results

### 3.1. Communication Capacity Evaluation with Multi-Hop Relaying

**Figure 11:**
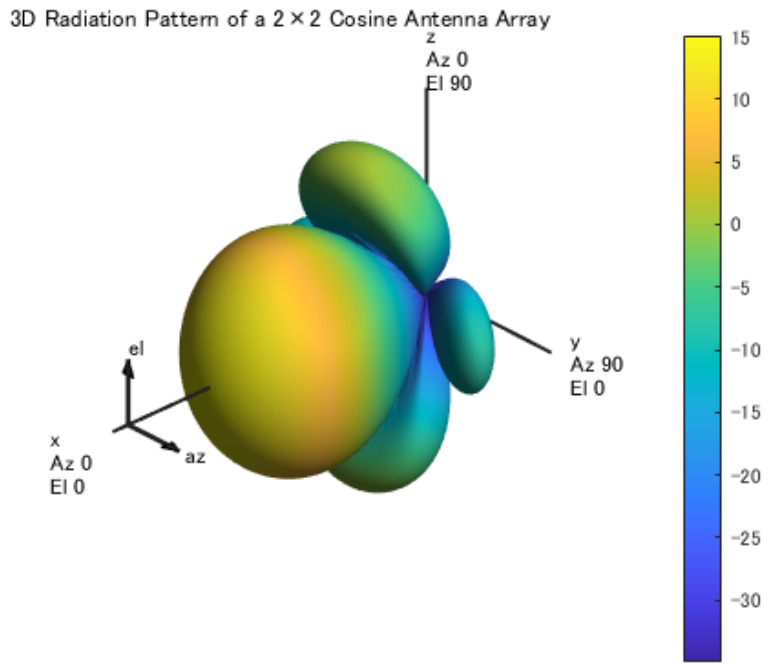
3D Antenna Pattern (2×2 Cosine Antenna Array).

In this section, we investigate the impact of beam control using high-gain and highly directional 2×2 patch antennas on the communication capacity of a multi-hop UAV backhaul network. In particular, we aim to clarify how the link distance reduction achieved by increasing the number of hops and the beam control effect enabled by directional antennas contribute to the bottleneck communication capacity of the overall network. In this study, a network configuration in which all UAVs are equipped with 2×2 patch antennas (antenna gain: 15.08 dBi) is considered, and the placement of relay UAVs is optimized using Particle Swarm Optimization (PSO). It should be noted that employing a full electromagnetic patch antenna model significantly increases the computational complexity of the optimization process. Since the present study involves large-scale placement optimization with repeated capacity evaluations, such a detailed model results in impractically long computation times. To represent highly directional antennas in millimeter-wave UAV backhaul communications, a 2×2 antenna array model is employed in this study. The antennas are assumed to operate in the 60 GHz millimeter-wave band targeted in this research. Each antenna element is implemented as a cosine-type antenna model using MATLAB’s hased. CosineAntennaElement. The radiation pattern is configured with CosinePower = [2, 2], which represents the directional characteristics in both the azimuth and elevation directions. This antenna model is adopted to efficiently perform network-level simulations in urban environments. Instead of using a detailed electromagnetic antenna structure, a mathematical antenna pattern that approximates the directional characteristics is used in order to maintain computational efficiency. Therefore, to ensure computational efficiency, a mathematical antenna model that approximates the directional characteristics of a 2×2 patch array in Figure 11 is adopted. This simplified model preserves the essential high-directivity behavior while enabling tractable optimization within a realistic computation time. The communication capacity evaluated in this section corresponds to the minimum link capacity, which determines the bottleneck performance of the multi-hop network. The simulation parameters used in this section are summarized in Table 1.

**Figure 12:**
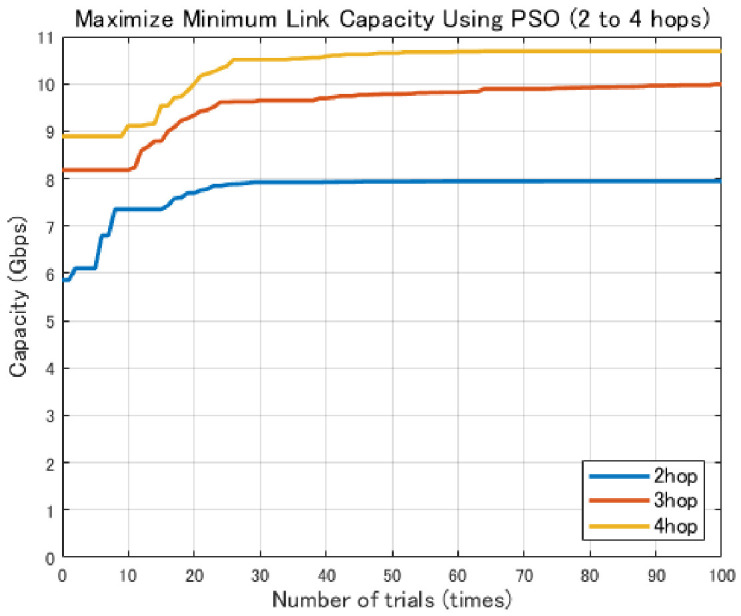
Comparison of Communication Capacity by Number of Hops.

Figure 12 shows the variation in the minimum link communication capacity for different numbers of hops when 2×2 patch antennas are employed. In this evaluation, the objective is to identify the number of hops required to achieve the target backhaul communication capacity of 9 Gbps per link, which is defined based on an assumed urban disaster scenario. Accordingly, multi-hop configurations with two to four hops are evaluated.

As shown in Figure 12, the minimum link communication capacity increases stepwise as the number of hops increases. In particular, a significant improvement in communication capacity is observed when moving from the two-hop to the three-hop configuration. This improvement can be attributed to the reduction in free-space path loss resulting from shorter link distances. Furthermore, the four-hop configuration also achieves a stable and high communication capacity, indicating that a multi-hop structure combined with high-gain antennas is effective for high-capacity backhaul communications.

**Figure 13:**
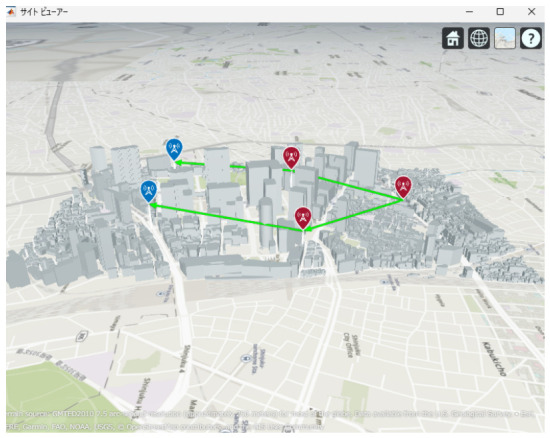

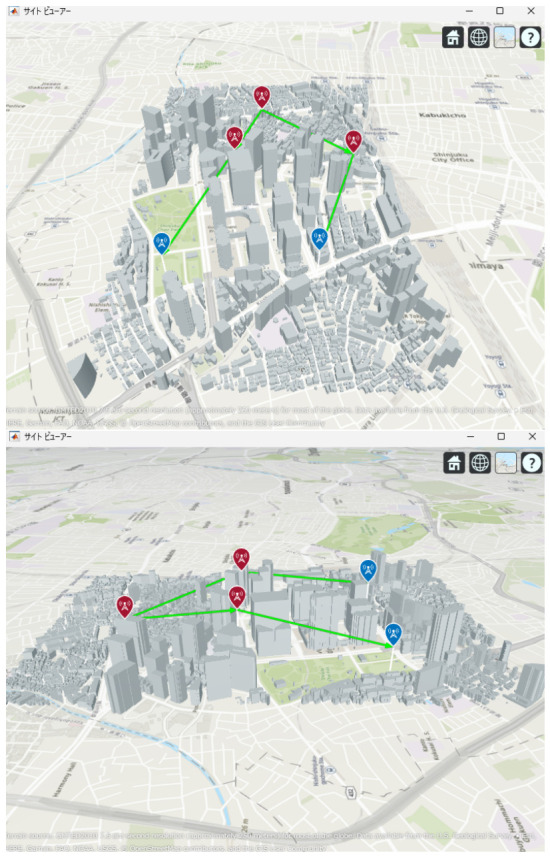
Optimal UAV Placement for 2 hop Communication.

**Figure 14:**
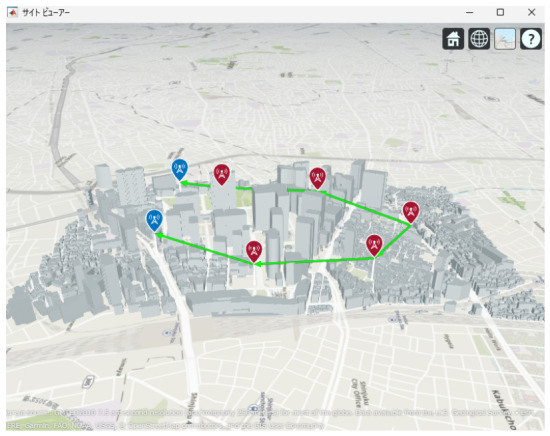

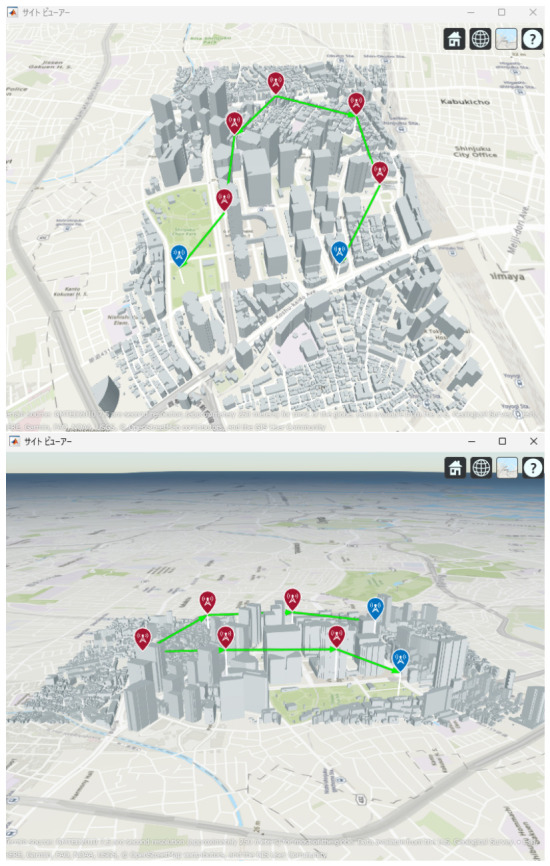
Optimal UAV Placement for 3 hop Communication.

**Figure 15:**
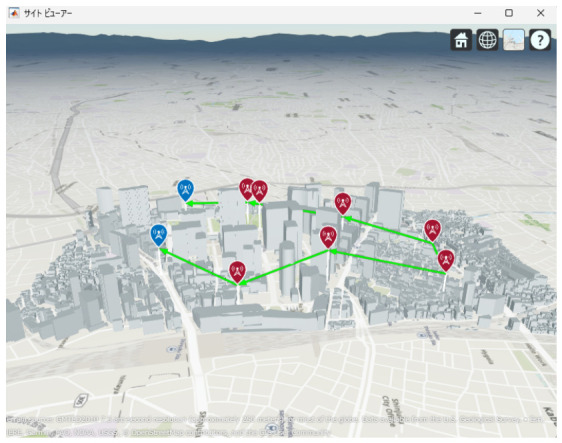

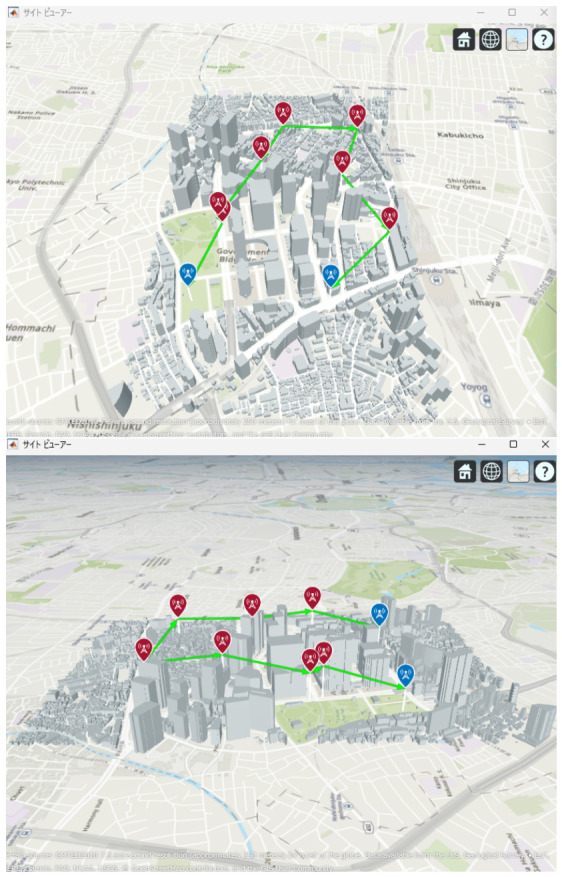
Optimal UAV Placement for 4-hop Communication.

Figure 13, Figure 14 and Figure 15 illustrate the optimized UAV placements for each hop configuration. Links are shown in green when LoS is established, and in red when LoS is not established. Although the relay UAVs are not necessarily placed at equal intervals, it can be observed that, in all configurations, they are positioned so as to avoid building blockage and maintain line-of-sight (LoS) conditions. This result indicates that PSO autonomously searches for UAV placements that simultaneously satisfy LoS availability and communication capacity maximization.

In addition, the 2×2 patch antenna enables high-gain and highly directional beamforming, allowing effective power concentration toward the main beam direction for each link. As a result, even when the number of relay UAVs increases, the impact of interference from surrounding links is relatively suppressed. Consequently, the benefit of link distance reduction is effectively reflected as an improvement in the signal-to-interference-plus-noise ratio (SINR).

From these results, it is demonstrated that in a multi-hop UAV backhaul network employing 2×2 patch antennas, the distance reduction effect obtained by increasing the number of hops and the beam control effect provided by directional antennas act synergistically to significantly enhance the communication capacity. Furthermore, the results reveal that achieving the target backhaul communication capacity of approximately 9 Gbps requires at least three hops in the considered scenario. In particular, the ability to achieve the target backhaul communication capacity on the order of 9 Gbps with a small number of hops is highly advantageous from a practical network design perspective, as it helps suppress increases in UAV operational cost and communication latency.

Therefore, in millimeter-wave multi-hop UAV backhaul networks, it is not sufficient to simply increase the number of relay UAVs. Instead, combining appropriate beam control using high-gain antennas with UAV placement optimization tailored to such beam characteristics is essential for realizing high-capacity and efficient communication networks.

### 3.2. Evaluation Under UAV Jitter

In this section, we evaluate how the minimum link capacity of the backhaul network varies when UAVs experience positional fluctuations caused by external factors such as wind, assuming realistic operational conditions. In practical environments, it is difficult for UAVs to maintain a perfectly stationary state, and even during hovering, positional deviations may occur in both horizontal and vertical directions. Therefore, in this study, random jitter is applied to the optimal UAV placements obtained in the previous chapter, and the resulting variations in communication capacity are quantitatively evaluated to clarify the impact of UAV jitter on communication performance.

#### 3.2.1. Evaluation Conditions

This subsubsection describes the UAV platform, jitter model, and simulation conditions used to evaluate the impact of UAV jitter on backhaul link capacity.

According to the operational guidelines for safe UAV flights issued by the Ministry of Land, Infrastructure, Transport and Tourism (MLIT) [28], safe flight cannot be ensured under wind speeds exceeding 5.0 m/s. Therefore, such conditions are excluded from consideration in this study. Under this assumption, UAV jitter is expected to remain within a range that still allows stable hovering.

Based on this premise, we assume positional deviations of ±1.5m in the horizontal direction and ±0.5m in the vertical direction during hovering. These values model realistic positioning errors that may occur due to wind effects in operational environments with wind speeds below 5.0 m/s.

These values are determined with reference to the hovering accuracy of the UAV platform assumed in this evaluation under wind conditions below 5.0 m/s.

In addition, experimental and numerical studies on mmWave UAV communications, such as the work by Masaoka et al. [29], have demonstrated the feasibility of UAV-based aerial base stations and highlighted that UAV motion and positional fluctuations can affect radio propagation characteristics. These findings further support the necessity of incorporating UAV positional jitter into the performance evaluation of UAV backhaul networks.

#### 3.2.2. UAV Platform

**Figure 16:**
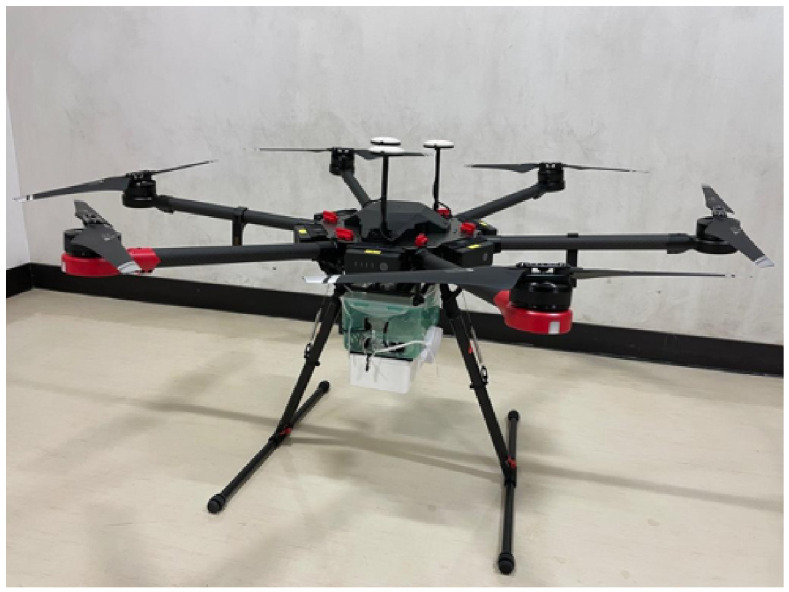
DJI Matrice 600 Pro [29].

The UAV platform assumed in this evaluation is the DJI Matrice 600 Pro, as shown in Figure 16. The Matrice 600 Pro is a large multirotor UAV capable of carrying multiple batteries, offering high payload capacity and stable hovering performance. In addition, it is rated to withstand wind speeds of up to approximately 8m/s, making it suitable for UAV-based backhaul relay applications [30].

To ensure a realistic evaluation, simulations are conducted using the UAV specifications published by DJI, as summarized in Table 2.

#### 3.2.3. Evaluation Procedure

The evaluation procedure adopted in this study is summarized as follows.

(1) A ray-tracing-based radio propagation simulation is constructed in MATLAB using a three-dimensional urban building model of the Shinjuku metropolitan area obtained from PLATEAU.
(2) One ground base station (BS) and two access UAVs are deployed at fixed positions, and a multi-hop backhaul network, including relay UAVs, is configured.
(3) While varying the positions of the backhaul UAVs, the communication capacity of each link is calculated, and the minimum link capacity of the entire backhaul network is evaluated.
(4) The positions of the backhaul UAVs are optimized to maximize the minimum link capacity, thereby determining the optimal UAV deployment under urban conditions.
(5) Based on the optimal UAV configuration obtained in the previous chapter, random jitter is applied to each UAV within ±1.5m horizontally and ±0.5m vertically in each trial.
(6) Communication capacity is evaluated after jitter is applied, taking into account beam misalignment effects, and the impact of UAV jitter on capacity degradation is analyzed.

Through this procedure, the influence of UAV jitter on communication capacity is evaluated for optimal UAV deployments in urban environments. In this section, we focus on capacity variations caused by jitter applied to the optimal configurations obtained in the previous chapter.

### 3.3. Capacity Evaluation Considering UAV Jitter

**Figure 17:**
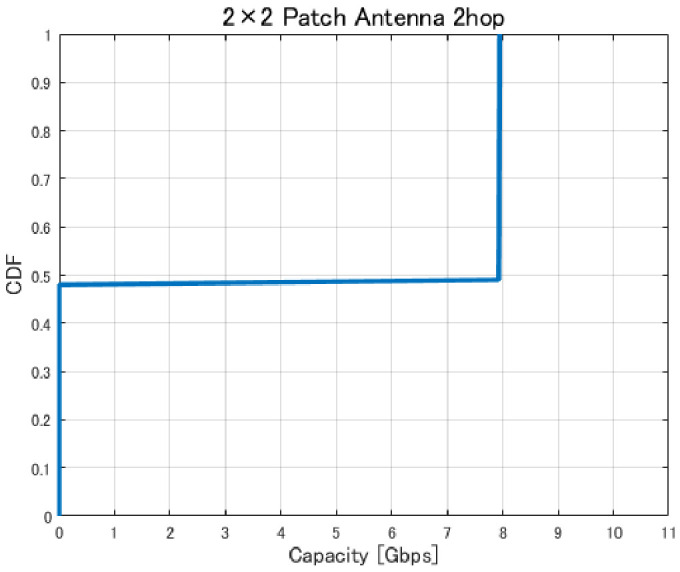
CDF of the minimum link capacity under UAV jitter (2 hops).

**Figure 18:**
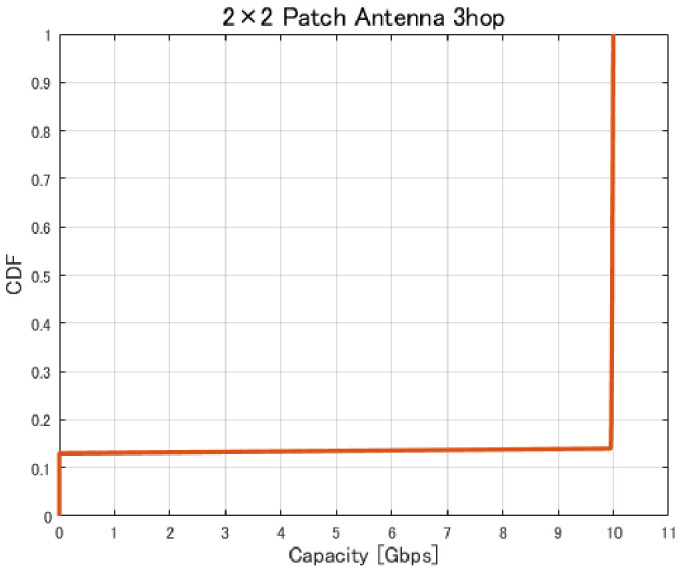
CDF of the minimum link capacity under UAV jitter (3 hops).

**Figure 19:**
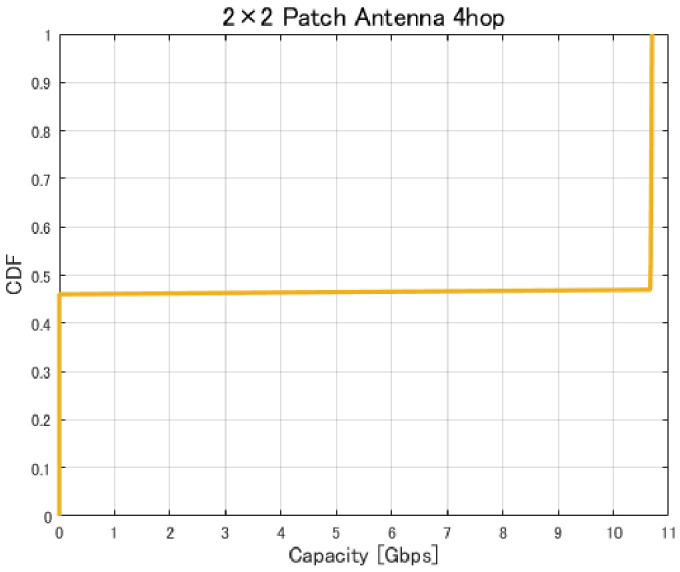
CDF of the minimum link capacity under UAV jitter (4 hops).

**Figure 20:**
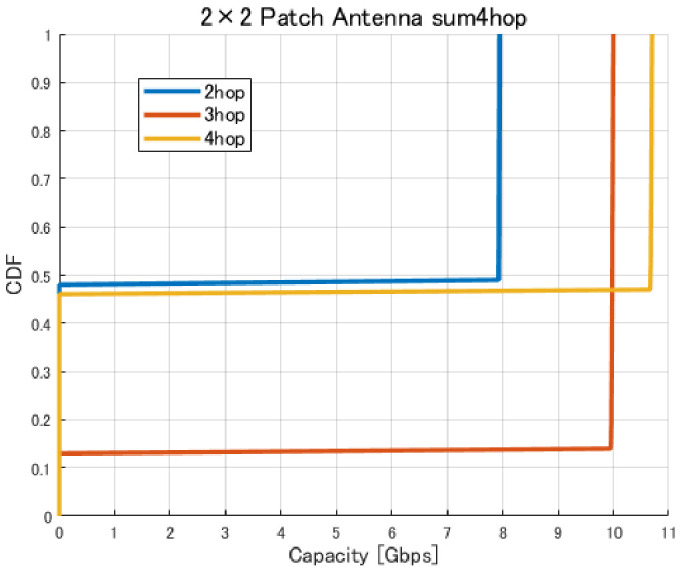
CDF of the minimum link capacity under UAV jitter (2–4 hops).

Figure 17, Figure 18, Figure 19 and Figure 20 show the cumulative distribution functions (CDFs) of the minimum link capacity when UAV jitter is considered, for 2–4-hop configurations using a 2×2 patch antenna.

**Figure 21:**
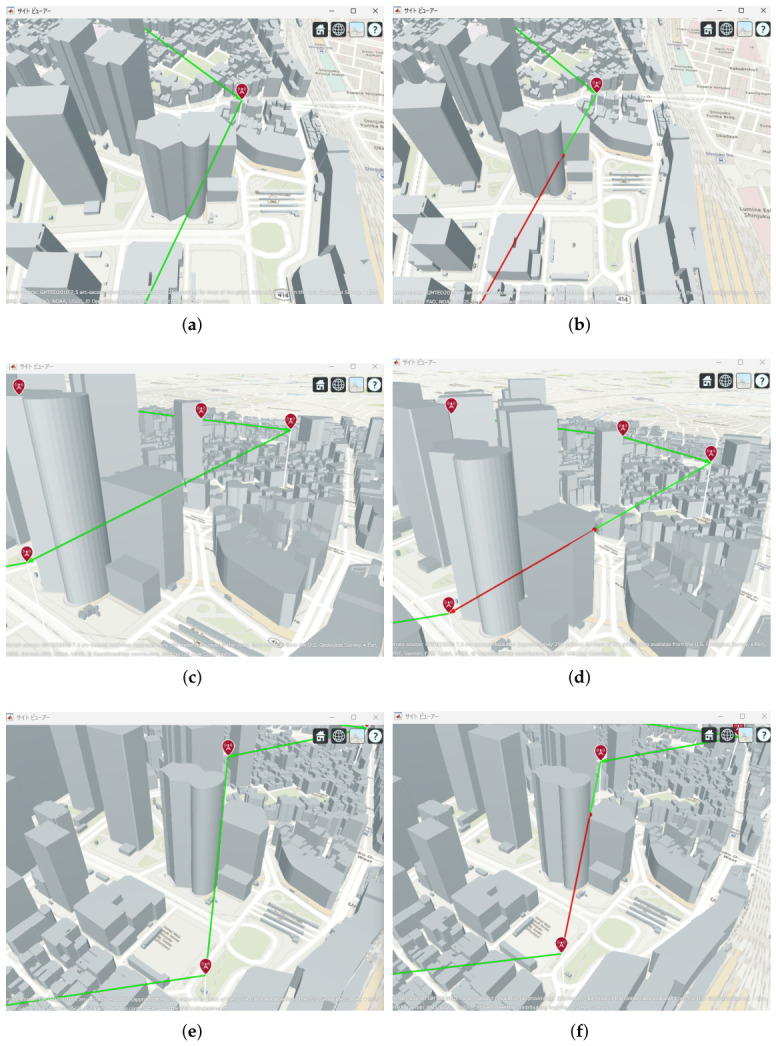
Comparison between the no-jitter optimal placement and the placement experiencing the maximum capacity degradation due to UAV jitter: (**a**) 2 hops without jitter (optimized deployment); (**b**) 2 hops with jitter (worst case). (**c**) 3 hops without jitter (optimized deployment); (**d**) 3 hops with jitter (worst case). (**e**) 4 hops without jitter (optimized deployment); (**f**) 4 hops with jitter (worst case).

The results indicate that even configurations achieving sufficient capacity under static conditions may experience severe capacity degradation once jitter is introduced. Detailed analysis reveals that small positional deviations of UAVs can cause beam misalignment, leading to blockage by buildings in urban environments and resulting in the loss of line-of-sight (LoS) conditions. Figure 21 illustrates examples of UAV deployments before and after jitter is applied, highlighting cases where capacity degradation is most severe. When the propagation path is obstructed by buildings due to UAV positional deviations, the LoS link may be lost, and the communication link may temporarily transition to a non-line-of-sight (NLoS) condition. Since millimeter-wave signals are highly sensitive to blockage, such transitions significantly reduce the received signal power, which in turn dominantly decreases the minimum link capacity of the entire backhaul network. These results demonstrate that UAV configurations optimized solely for static conditions are not necessarily robust against jitter in practical operations, particularly in dense urban environments where UAVs may be deployed close to buildings.

### 3.4. Jitter-Aware Position Optimization Method

To address the above issue, we propose a jitter-aware position optimization method. In the proposed method, UAV jitter is explicitly considered during the optimization process, and the UAV deployment is searched such that line-of-sight (LoS) conditions can be maintained even when jitter occurs.

**Figure 22:**
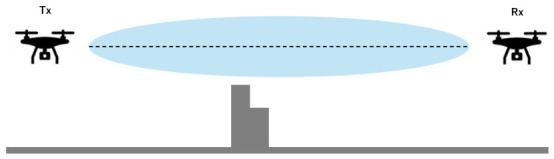
Illustration of a Fresnel zone.

First, we describe the Fresnel zone used in this study. The Fresnel zone is a rotational ellipsoidal region around the line connecting the transmitter and receiver, representing the space that should be kept clear to ensure efficient radio propagation. Among the Fresnel zones, the first Fresnel zone has the greatest impact on radio propagation, and the intrusion of obstacles into this region may cause diffraction and blockage, resulting in signal attenuation and degradation of communication quality. The relationship between the Fresnel zone and obstacles is illustrated in Figure 22.

If obstacles such as buildings intrude into the Fresnel zone, the received power can be reduced due to diffraction and blockage effects, which degrades communication quality [31].

Let the distance between the transmitter and receiver be $d_{1}+d_{2}$, and the wavelength be λ. The radius of the *n*-th Fresnel zone, denoted by $R_{n}$, is given by

(8) $$R_{n}=\sqrt{\frac{n\lambda d_{1}d_{2}}{d_{1}+d_{2}}}.$$

In this study, we focus on the first Fresnel zone, i.e., n=1.

Next, we explain how the Fresnel zone is handled under UAV jitter. In this study, rather than performing a stochastic time-series simulation of UAV motion, we introduce a conservative geometric margin into the Fresnel-zone evaluation so that UAV placements robust against expected position deviations can be selected. Specifically, we assume hovering jitter within ±1.5m in the horizontal direction and ±0.5m in the vertical direction. Due to UAV jitter, the positions of both the transmitter and receiver may vary, and thus the distances $d_{1}$ and $d_{2}$ from an evaluation point can increase by up to 1.5m, respectively.

Therefore, we consider a worst-case geometry where the relative distances become $d_{1}+1.5\;m$ and $d_{2}+1.5\;m$, and we evaluate the Fresnel zone radius under this condition. Specifically, we define the jitter-aware *n*-th Fresnel zone radius $R_{n}^{\mathrm{jitter}}$ as

(9) $$R_{n}^{\mathrm{jitter}}=\sqrt{\frac{n\lambda \left(d_{1}+1.5\right)\left(d_{2}+1.5\right)}{\left(d_{1}+1.5\right)+\left(d_{2}+1.5\right)}}.$$

Furthermore, to include an additional safety margin against position fluctuations, we add the maximum horizontal deviation 1.5m to the above radius, and define the final evaluation radius *R* as

(10) $$R=R_{n}^{\mathrm{jitter}}+1.5\;m.$$

In our simulations, the proposed method imposes a constraint that the expanded Fresnel zone does not intersect with obstacles such as buildings. More specifically, on the plane orthogonal to the LoS path, four representative offset lines, denoted by ±v and ±w, are constructed at a distance corresponding to the evaluation radius *R*. LoS blockage is then checked for all of these four offset lines. A link is regarded as satisfying the Fresnel-zone constraint only when all four offset lines remain unobstructed.

By enforcing this constraint during optimization, we search for robust UAV deployments that can maintain LoS conditions even under UAV jitter. Thus, the proposed method does not directly simulate every instantaneous UAV displacement, but instead adopts a conservative clearance test to ensure robustness against expected UAV position deviations.

#### Evaluation Procedure Considering the Fresnel Zone

**Figure 23:**
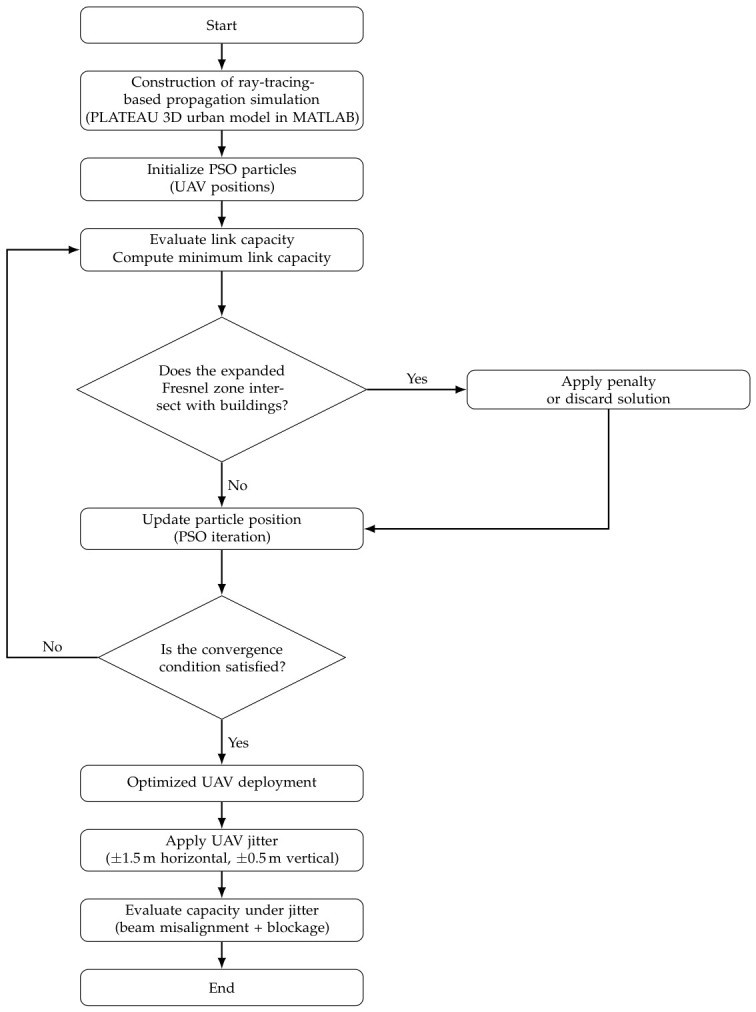
Flowchart of jitter-aware UAV placement optimization with Fresnel-zone constraint.

The evaluation procedure based on the proposed method is described below. In correspondence with the processing flow shown in Figure 23, we sequentially conduct radio propagation evaluation in an urban environment, jitter-aware position optimization, and capacity evaluation under jitter.

Through the above procedure, we aim to construct a robust UAV backhaul network that can suppress performance degradation even when UAV jitter occurs.

### 3.5. Performance Improvement by the Proposed Method

**Figure 24:**
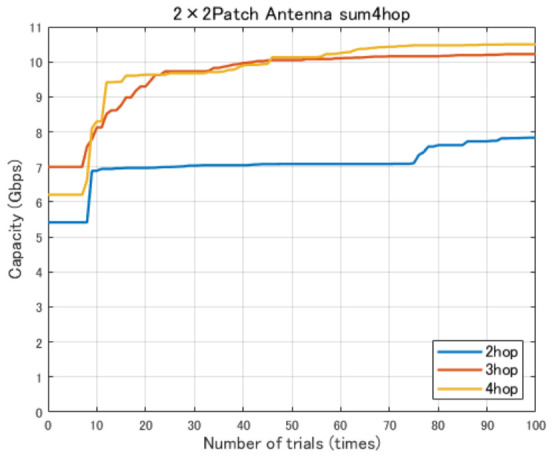
Evolution of the minimum link capacity in jitter-aware position optimization with Fresnel-zone constraint (2–4 hops).

Figure 24 shows the evolution of the minimum link capacity obtained by the proposed method with the Fresnel-zone constraint. By imposing the constraint that the expanded Fresnel zone does not intersect buildings, the optimization process explicitly searches for UAV deployments that are less likely to be blocked even when jitter occurs. As a result, for all hop configurations, the solutions are gradually improved with increasing iterations and converge to stable, high minimum link capacities.

**Figure 25:**
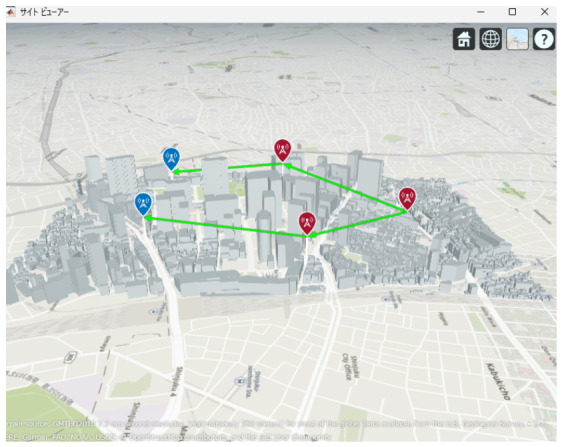

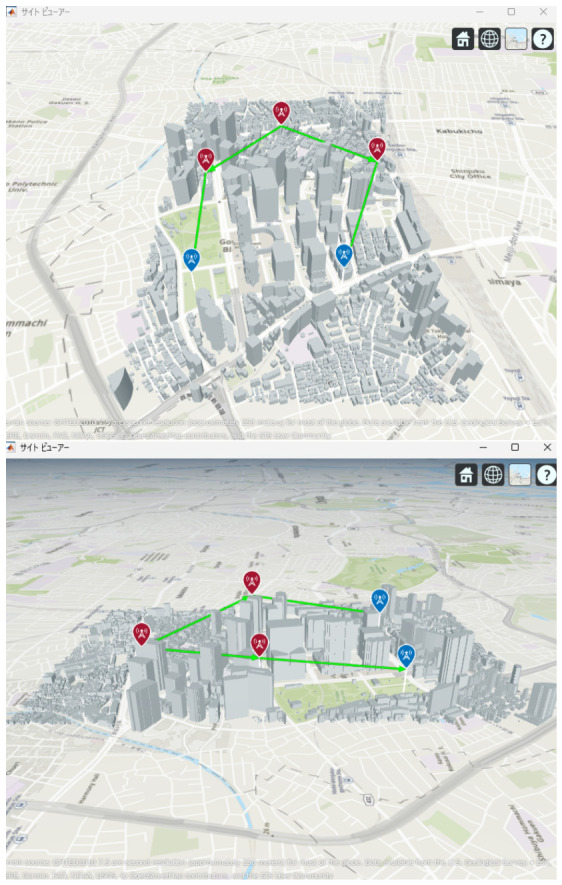
Optimal UAV Placement for 2-hop Communication with Fresnel Zone Consideration.

**Figure 26:**
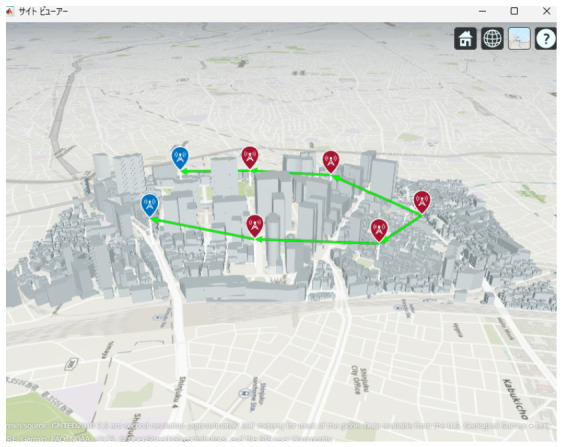

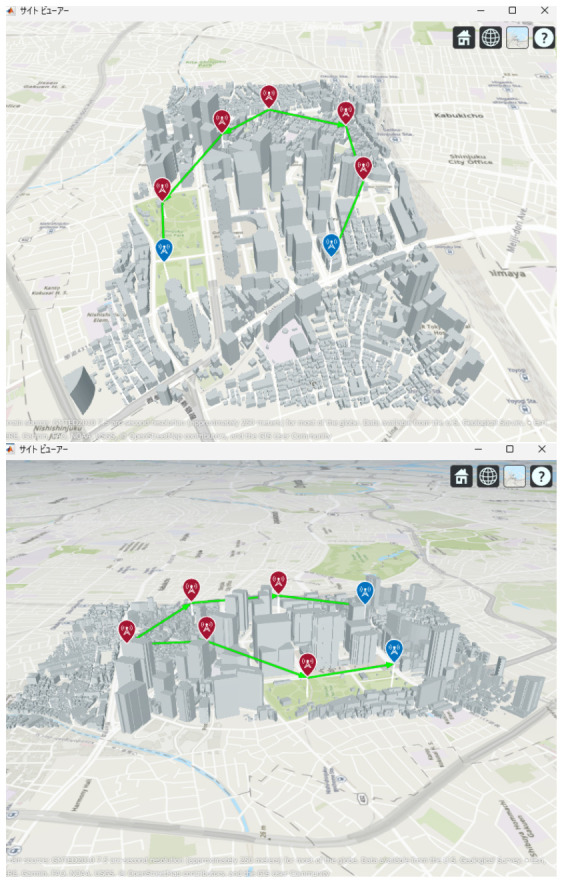
Optimal UAV Placement for 3-hop Communication with Fresnel Zone Consideration.

**Figure 27:**
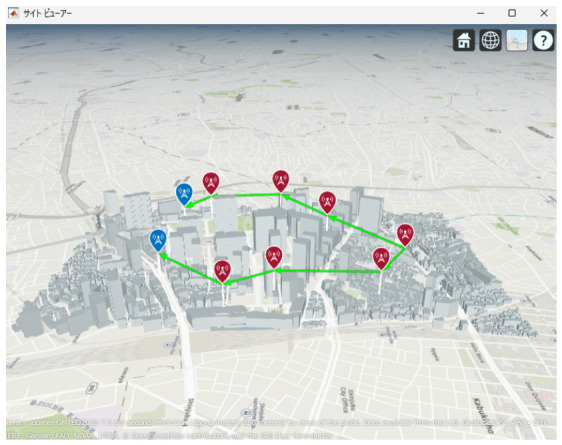

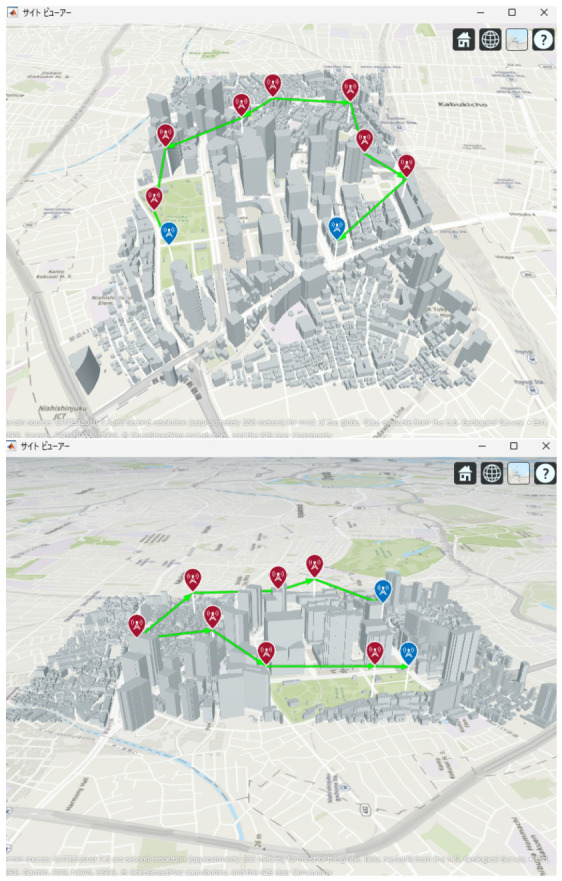
Optimal UAV Placement for 4-hop Communication with Fresnel Zone Consideration.

Next, the optimal UAV deployments obtained by the proposed method are shown in Figure 25, Figure 26 and Figure 27. Each figure presents the optimized UAV deployment for 2-hop, 3-hop, and 4-hop configurations from three viewpoints: East view, Top view, and West view. In all hop configurations, relay UAVs are placed to avoid buildings while ensuring sufficient spatial clearance for the Fresnel zone, which makes the LoS condition more likely to be maintained under UAV jitter.

**Figure 28:**
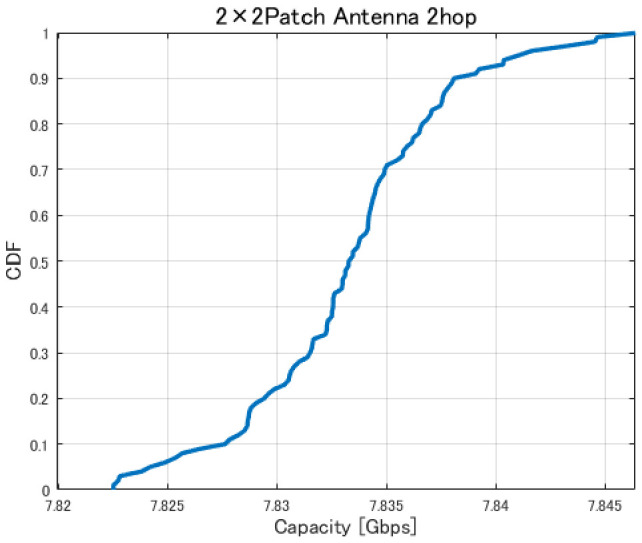
CDF of the minimum link capacity under UAV jitter for the proposed deployment (2 hops).

**Figure 29:**
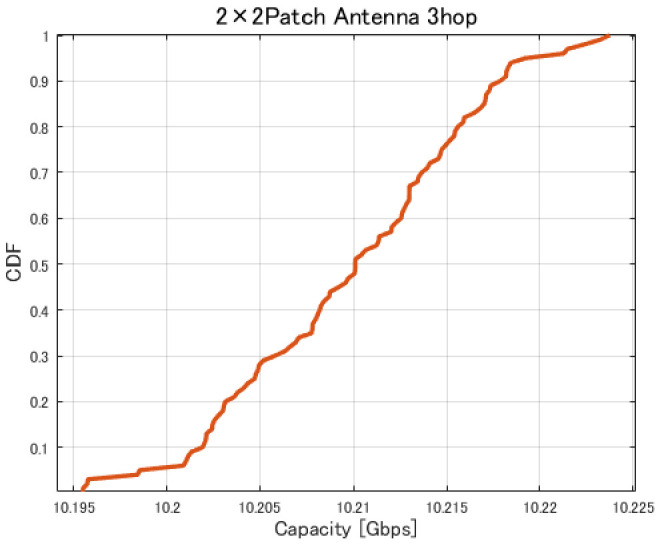
CDF of the minimum link capacity under UAV jitter for the proposed deployment (3 hops).

**Figure 30:**
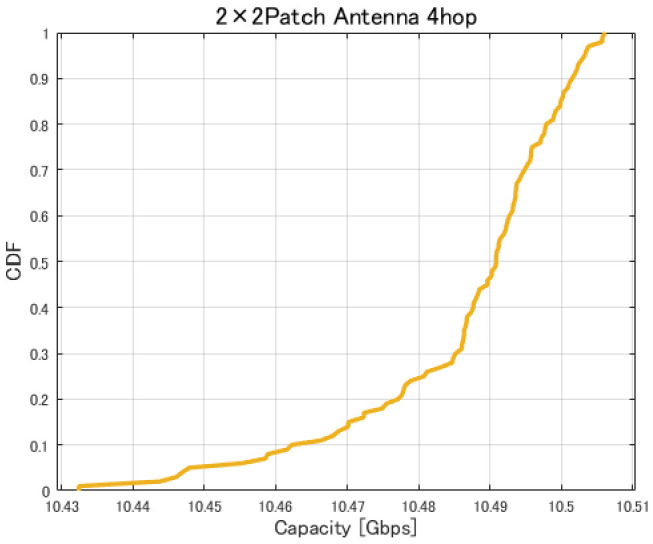
CDF of the minimum link capacity under UAV jitter for the proposed deployment (4 hops).

**Figure 31:**
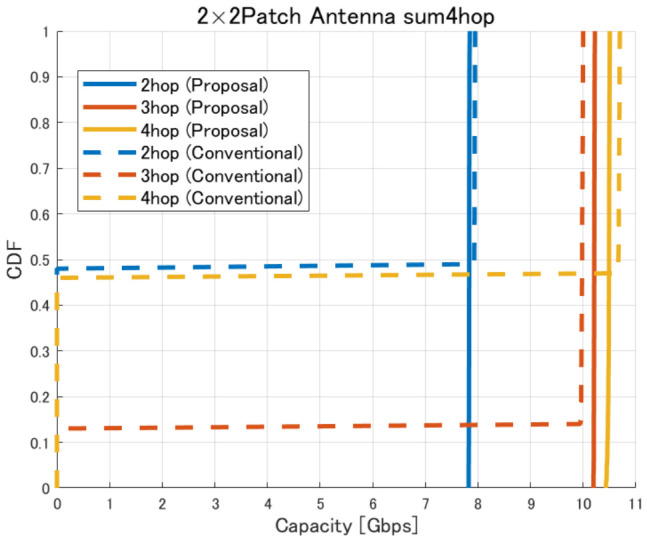
CDF comparison of the minimum link capacity under UAV jitter for the proposed deployment (2–4 hops).

Furthermore, Figure 28, Figure 29, Figure 30 and Figure 31 show the CDFs of the minimum link capacity when UAVs are randomly jittered within ±1.5m horizontally and ±0.5m vertically based on the optimized deployments obtained by the proposed method.

**Figure 32:**
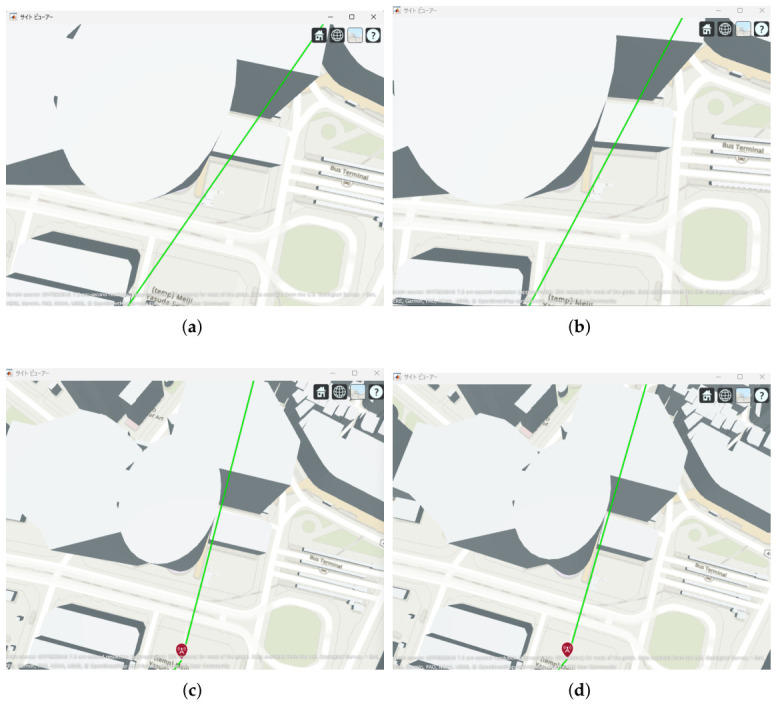

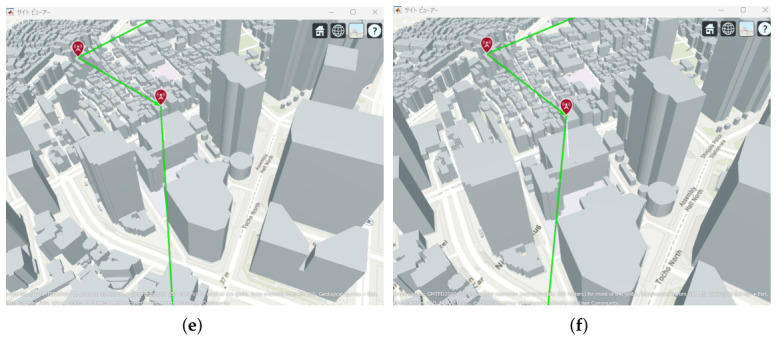
Comparison between the no-jitter optimal placement and the placement experiencing the maximum capacity degradation due to UAV jitter, considering the Fresnel zone: (**a**) 2 hops without jitter (optimized deployment); (**b**) 2 hops with jitter (worst case); (**c**) 3 hops without jitter (optimized deployment); (**d**) 3 hops with jitter (worst case); (**e**) 4 hops without jitter (optimized deployment); (**f**) 4 hops with jitter (worst case).

The results indicate that, in the conventional method without the Fresnel-zone constraint, as shown in Figure 20, UAV jitter can cause building blockage and LoS loss on one or more links, which may lead to significant degradation of communication capacity. In contrast, in the proposed method, as shown in Figure 31, the degradation of communication capacity tends to be suppressed even when UAV jitter occurs. This is because sufficient spatial clearance, including the Fresnel zone, is ensured during optimization so that even if beam directions change due to small UAV position deviations, the main beam is less likely to be blocked by buildings, as illustrated in Figure 32. Furthermore, it was confirmed that the three-hop UAV deployment required to achieve the target backhaul communication capacity of approximately 9 Gbps in the proposed method also exhibits strong robustness against UAV jitter. This result indicates that the UAV placements obtained by considering the Fresnel-zone constraint not only achieve high communication capacity but also maintain stable link conditions even under practical UAV position fluctuations.

Furthermore, from the perspective of quantitative metrics such as outage probability, confidence interval, and average capacity loss, it is confirmed that the proposed method effectively suppresses the degradation of communication performance under UAV jitter conditions. Specifically, as shown in Figure 20, in the conventional method, UAV jitter increases the probability that the capacity falls below a required threshold (outage probability). For example, for a target capacity of 9 Gbps, the outage probability reaches approximately 15% and 48% for the three-hop and four-hop configurations, respectively. In addition, the CDF shape indicates that the capacity is distributed over a wide range, confirming that performance fluctuations due to jitter are significant. Furthermore, from the viewpoint of the confidence interval, the conventional method exhibits large variability in capacity, leading to high uncertainty in the estimated average capacity and suggesting that link quality is not stable. In contrast, as shown in Figure 31, in the proposed method, the average capacity loss is limited to approximately 0.02–0.03 Gbps for the two-hop case, about 0.03 Gbps for the three-hop case, and about 0.07 Gbps for the four-hop case. At the same time, the outage probability is reduced to 0% for both the three-hop and four-hop configurations. Furthermore, since the capacity distribution is concentrated around a specific value, it is confirmed that performance variability under jitter conditions is significantly suppressed. In addition, the confidence interval tends to be narrower, indicating improved estimation accuracy of the average capacity and statistically more stable communication performance. These results quantitatively demonstrate that the proposed method achieves high robustness against UAV jitter and provides stable communication performance.

In this study, the target capacity of 9 Gbps was set assuming a disaster communication scenario in a dense urban area such as Shinjuku Ward, Tokyo. However, the proposed UAV placement optimization method itself is not dependent on a specific communication capacity target. Therefore, even when different target capacities are required depending on communication demand or network conditions, the proposed method can be applied in the same manner.

## 4. Conclusions

In this study, we investigated the improvement of communication capacity by introducing directional antennas into UAV placement optimization based on Particle Swarm Optimization (PSO), explicitly taking antenna gain into account. Furthermore, by applying a multi-hop configuration using multiple UAVs, we demonstrated that the backhaul communication capacity can be significantly enhanced compared to a single-hop configuration.

In addition, from the perspective of reliability, we evaluated the impact of UAV position fluctuations on communication capacity. The results confirm that UAV oscillations can cause antenna beams to overlap with surrounding buildings, leading to a significant degradation in communication capacity.

This study does not aim to establish a novel theoretical framework; rather, it is based on existing theories and introduces a heuristic design that takes into account practical factors such as UAV jitter and building blockage and proposes a UAV placement optimization method that ensures sufficient Fresnel zone clearance. The results confirm that although communication capacity inevitably fluctuates due to UAV oscillations, the proposed method effectively suppresses severe degradation in communication quality even under realistic oscillation conditions assumed for practical deployment.

## Figures and Tables

**Table 1:** Simulation Parameters for Multi-Hop Communication Evaluation.

Parameter	Value
Carrier Frequency	60 GHz
Bandwidth	2.16 GHz
Maximum Number of Reflections	0
Maximum Number of Diffractions	0
Simulation Area	Shinjuku City Hall surroundings
Longitude Range	139.687500° E–139.699998° E
Latitude Range	35.683330° N–35.699999° N
Backhaul UAV Altitude (Minimum)	50 m
Backhaul UAV Altitude (Maximum)	150 m
UAV Transmit Power	24 dBm
2×2 Patch Antenna Gain	15.08 dBi
Base Station Location (Lat., Lon., Antenna height.)	(35.697028° N, 139.693400° E, 3 m)
Access UAV AC-1 Location (Lat., Lon., Alt.)	(35.687373° N, 139.689739° E, 100 m)
Access UAV AC-2 Location (Lat., Lon., Alt.)	(35.687417° N, 139.695891° E, 110 m)

**Table 2:** Specifications of the DJI Matrice 600 Pro.

Model Name	Matrice 600 Pro
Manufacturer	DJI
Overall Dimensions	1668 mm × 1518 mm × 727 mm
Weight (Without Payload)	9.5 kg
Maximum Takeoff Weight	15.5 kg
Maximum Wind Resistance	8 m/s
Maximum Hovering Time	32 min
Battery Capacity	4500 mAh
Battery Voltage	22.2 V
Number of Batteries	6
Remote Controller Operating Frequency	920.6–928 MHz
Maximum Transmission Distance	3.5 km

## Data Availability

The original contributions presented in this study are included in the article. Further inquiries can be directed to the corresponding authors.
